# Genomic and Transcriptomic Landscape and Evolutionary Dynamics of Heat Shock Proteins in Spotted Sea Bass (*Lateolabrax maculatus*) under Salinity Change and Alkalinity Stress

**DOI:** 10.3390/biology11030353

**Published:** 2022-02-23

**Authors:** Xujian Li, Saisai Liu, Yapeng Wang, Wei Lu, Quanqi Zhang, Jie Cheng

**Affiliations:** 1 Key Laboratory of Marine Genetics and Breeding, Ministry of Education, Ocean University of China, 5 Yushan Road, Qingdao 266003, China; lxj1316@stu.ouc.edu.cn (X.L.); liusaisai@stu.ouc.edu.cn (S.L.); wangyapeng@stu.ouc.edu.cn (Y.W.); lw1981@ouc.edu.cn (W.L.); qzhang@ouc.edu.cn (Q.Z.); 2 Laboratory for Marine Fisheries Science and Food Production Processes, Pilot National Laboratory for Marine Science and Technology, 1 Wenhai Road, Qingdao 266237, China; 3 Key Laboratory of Tropical Aquatic Germplasm of Hainan Province, Sanya Oceanographic Institution, Ocean University of China, Sanya 572024, China

**Keywords:** heat shock protein, co-chaperon network, salinity-alkalinity adaptation, molecular evolution, *Lateolabrax maculatus*

## Abstract

**Simple Summary:**

Heat shock proteins (Hsps) are ubiquitous and conserved in almost all living organisms and are involved in a wide spectrum of cellular responses against diverse environmental stresses. However, our knowledge about the coordinated Hsp co-chaperon interaction is still limited, especially in aquatic animals facing dynamic water environments. In this study, we provided the systematic analysis of 95 *Hsp* genes (*LmHsps*) in spotted sea bass (*Lateolabrax maculatus*), an important aquaculture species in China, under salinity change and alkalinity stress through in silico analysis. The coordinated expression of *LmHsp*s in response to salinity change and alkalinity stress in the gills was determined. Our results confirmed the diverse regulated expression of *Hsp*s in *L. maculatus*, and that the responses to alkalinity stress may have arisen through the adaptive recruitment of *LmHsp40*-*70*-*90* co-chaperons. Our results provide vital insights into the function and adaptation of aquatic animal Hsps in response to salinity-alkalinity stress.

**Abstract:**

The heat shock protein (Hsp) superfamily has received accumulated attention because it is ubiquitous and conserved in almost all living organisms and is involved in a wide spectrum of cellular responses against diverse environmental stresses. However, our knowledge about the Hsp co-chaperon network is still limited in non-model organisms. In this study, we provided the systematic analysis of 95 *Hsp* genes (*LmHsp*s) in the genome of spotted sea bass (*Lateolabrax maculatus*), an important aquaculture species in China that can widely adapt to diverse salinities from fresh to sea water, and moderately adapt to high alkaline water. Through in silico analysis using transcriptome and genome database, we determined the expression profiles of *LmHsp*s in response to salinity change and alkalinity stress in *L. maculatus* gills. The results revealed that *LmHsp*s were sensitive in response to alkalinity stress, and the *LmHsp40*-*70*-*90* members were more actively regulated than other *LmHsp*s and may also be coordinately interacted as co-chaperons. This was in accordance with the fact that members of *LmHsp40*, *LmHsp70*, and *LmHsp90* evolved more rapidly in *L. maculatus* than other teleost lineages with positively selected sites detected in their functional domains. Our results revealed the diverse and cooperated regulation of *LmHsp*s under alkaline stress, which may have arisen through the functional divergence and adaptive recruitment of the *Hsp40*-*70*-*90* co-chaperons and will provide vital insights for the development of *L. maculatus* cultivation in alkaline water.

## 1. Introduction

Heat shock proteins (Hsps) are highly conserved stress proteins existing broadly from prokaryotes to mammals with chaperone activity [1,2], which play essential roles in maintaining the stability of cell structure and mitigating the effect of protein misfolding and aggregation in almost all aspects of protein metabolism [3]. The Hsp superfamily could be classified into Hsp10 (Hspe, 10 kDa), small Hsp (sHsp/Hspb, 20–30 kDa), Hsp40 (Dnaj, 40 kDa), Hsp60 (Hspd, 60 kDa), Hsp70 (Hspa, 70 kDa), Hsp90 (Hspc, 83~90 kDa), and Hsp100/110 (Hsph, 100–110 kDa) according to their relative molecular mass, structure homology, and function [4]. For example, sHsps can bind a large range of non-native substrate proteins to form a sHsp substrate complex prior to irreversible aggregation, which becomes the target of ATP-dependent Hsp70–Hsp100 disaggregation activity [5]. Hsp40s, also known as J-domain proteins (Dnajs), have a key role in the process of transferring substrates to their Hsp70 partners by stimulating the ATP hydrolysis of Hsp70 [6]. Hsp10/60s are co-chaperones mainly implicated in mitochondrial protein import and macromolecular assembly, which function together to facilitate the correct folding of imported proteins [7]. Hsp70s and Hsp90s are both ATP-dependent molecular chaperones, which perform numerous functions in a wide variety of cellular processes, including the protection of proteins from stresses [8]. In addition, the Hsp chaperons also cooperate with each other to constitute a dynamic and functionally versatile network for diverse cellular functions [9]. For instance, Hsp70 and Hsp40 could interact with a substrate protein and co-chaperones to facilitate the transfer of the substrate to Hsp90 [9,10], while Hsp90 and Hsp70 could also directly collaborate with each other [10]. In contrast, sHsps function independently of ATP so as to prevent the formation of large insoluble protein aggregates [11].

With the increasing impacts of climate change and anthropogenic stresses, the function of the heat-shock system affecting organisms’ evolutionary fitness in diverse environments is a growing and important topic [12]. A large number of studies have focused on *Hsp* families in animals living in a highly dynamic aquatic environment. For instance, changes in abiotic factors (temperature, salinity, pH, dissolved oxygen, heavy metals, or other pollutants) [13,14,15] as well as biotic pathogen and toxic algae infection [16,17,18,19,20] could cause stresses in aquatic animals. Accordingly, *Hsp*s are expressed significantly and act as chaperones to help cells maintain normal physiological activities related to growth, reproduction, and physiological homeostasis. In particular, salinity changes in aquatic environments can increase the lysozyme activity, mucosal production, and immune defense in teleosts, and high alkalinity is harmful to most fishes, leading to health damage and even degradation of biomolecules [21]. Recently, although numerous studies have strongly implicated a critical role of *Hsp*s in salinity-alkalinity adaptation [22,23,24], most of them are only focused on responses for a limited number of *Hsp*s rather than the full complement of genes that comprise the Hsp co-chaperone network in salinity-alkalinity adaptation. Therefore, with the saline-alkaline water aquaculture becoming a promising way to accommodate the growing need for the aquaculture industry, it is important to investigate the physiological change and molecular response in fishes adapting to saline-alkaline waters, which could also help to understand their adaptation in the aquatic ecosystem.

Spotted sea bass (*Lateolabrax maculatus*) is an economically important fish with a high nutritional value that is widely distributed in the coastal and estuarine areas of China, Japan, and the Korean peninsula [25]. *L. maculatus* has an excellent ability to adapt to a broad variety of salinity environments ranging from fresh to sea water [26]; therefore, its aquaculture is viable in both freshwater ponds and sea water cages in China. Additionally, *L. maculatus* has been proved to be able to survive in high alkaline water (carbonate alkalinity = 10 mmol/L) for a long period of time [27], and the aquaculture of *L. maculatus* in alkaline water has been under development recently. To explore the function of *Hsp* superfamily in *L. maculatus* (*LmHsp*) under salinity change and alkalinity stress, in this study, 95 *LmHsp* genes were identified in *L. maculatus* genome, and their phylogeny, conserved structure, expression profile, possible co-chaperon network under stress, as well as molecular evolution pattern were investigated. Our results provide comprehensive insights for further understanding the environmental adaptation of *Hsp* co-chaperons in *L. maculatus* and will help to establish the alkaline water aquaculture of *L. maculatus*.

## 2. Materials and Methods

### 2.1. Genome-Wide Identification and Annotation of Hsp Families in L. maculatus

To identify *LmHsp* genes, the coding sequences (cds) and amino acid (aa) sequences of *L. maculatus* genome (PRJNA408177) were blast searched using available *Hsp* cds and aa sequences from nine fish species, including zebrafish (*Danio rerio*), channel catfish (*Ictalurus punetaus*), stickleback (*Gasterosteus aculeatus*), medaka (*Oryzias latipes*), tilapia (*Oreochromis niloticus*), turbot (*Scophthalmus maximus*), fugu (*Takifugu rubripes*), Asian sea bass (*Lates calcarifer*), spotted gar (*Lepisosteus oculatus*), and four tetrapod species, including human (*Homo sapiens*), mouse (*Mus musculus*), chicken (*Gallus gallus*), and Xenopus (*Xenopus tropicalis*) from Ensemble (http://asia.ensembl.org accessed on 9 November 2021) and NCBI (http://www.ncbi.nlm.nih.gov accessed on 9 November 2021). These sequences were used as query to perform BLASTN and BLASTP (e-value = 1 × 10^−5^) against the sequence resources of *L. maculatus*. The conserved domains of candidate Hsp sequences were predicted using SMART [28] and Pfam [29], and the sequences without a corresponding complete Hsp domain were excluded from further analysis. When a gene could not be accurately distinguished, it was named after the zebrafish orthologs with letter “L” (meaning “like”).

For the identified LmHsp aa sequences, the Multiple Em for Motif Elicitation (MEME, https://meme-suite.org/meme accessed on 18 November 2021) program was applied to evaluate their conserved motifs, with the parameters of any number of repetitions, optimum width of motifs from 6 to 200 with 4 motifs for sHsp, 5 motifs for Hsp40, 3 motifs for Hsp10/60, 13 motifs for Hsp70, 6 motifs for Hsp90, and 3 motifs for Hsp100. The Gene Structure Display Sever (GSDS 2.0, http://gsds.gao-lab.org accessed on 20 November 2021) was used to visualize the exon-intron of *LmHsp* genes, and the diagrammatic sketches were illustrated using TBtools v1.09 [30].

### 2.2. Phylogenetic Analysis of Hsp Families

All Hsp aa sequences from *L. maculatus* and other selected vertebrates mentioned before were used to construct phylogenetic trees. Multiple protein sequence alignments were conducted with Muscle [31]. According to the Bayesian information criterion (BIC), the best models selected for phylogeny construction were JTT + F + G4 model for sHsp, VT + G4 model for Hsp40, LG + I + G4 model for Hsp60, JTT + G4 model for Hsp70, LG + G4 model for Hsp90, and JTT + I for Hsp100. The Maximum Likelihood (ML) phylogenetic trees of each Hsp family were generated using IQ-Tree [32], respectively, with a bootstrap of 1000 replicates. Furthermore, iTOL [33] was used to visualize the phylogenetic trees.

### 2.3. Expression of LmHsp Genes through Transcriptome Analysis

To investigate the expression profiles of *LmHsp* genes in *L. maculatus* tissues, transcriptome data of seven adult tissues were obtained from NCBI (brain-SRR7528887, stomach-SRR7528884, spleen-SRR7528888, liver-SRR7528886, gill-SRR7528883, testis-SRR7528885, and ovary-SRR2937376). Through the Hisat and StringTie pipeline [34], the Fragments Per Kilobase of exon per Million mapped reads (FPKM) of each *LmHsp* gene were obtained. To understand the expression dynamics of *LmHsp*s under stresses, the RNA-seq data was also obtained from NCBI as gill tissues under salinity change (PRJNA515986) and alkalinity stress (PRJNA611641), respectively. The former data represented *L. maculatus* specimens (158.23 ± 18.77 g) originated from sea water (13.5–14.5 °C, pH 7.8–8.15, salinity 30 ppt) with salinity acclimation in fresh water (0 ppt, control group), brackish water (15 ppt), and sea water (30 ppt) for 30 days, respectively [35]. The latter data represented *L. maculatus* specimens (body weight: 140.32 ± 2.56 g) acclimated in fresh water (pH 7.8–8.15) for 30 days (control group) and then challenged with alkalinity stress (carbonate alkalinity = 18 mmol/L with NaHCO_3_ and Na_2_CO_3_ added to fresh water) at 0 h, 12 h, 24 h, and 72 h [27]. All the experimental groups contained three individuals for biological replicate, which represented a good correlation among groups from principal component analysis (PCA) (Figure S1). These RNA-seq data were processed with the Hisat and StringTie pipeline and the log_2_ (fold change) (log_2_FC) for *LmHsp* genes between the test and control group was calculated by edgeR [36]. TBtools was employed to draw heatmaps with log_2_ (FPKM + 1) and log_2_FC values, respectively. In addition, the trend analysis of gene expression was conducted to cluster *LmHsp* genes with similar expression patterns along the test time points under alkalinity stress via online toolkit OmicShare (https://www.omicshare.com/ accessed on 17 Feburary 2022).

### 2.4. Protein-Protein Interaction and Co-Expression Analysis

Protein-protein interaction (PPI) networks among *Hsp* families were analyzed via the online STRING 11.5 tool (https://string-db.org/ accessed on 10 October 2021) for human and zebrafish, with the parameter minimum required interaction score of 0.7. For *L. maculatus Hsps* in response to alkalinity stress, the expression correlation between each *LmHsp* gene was evaluated using pairwise Pearson’s correlation coefficient (PCC) with their FPKM and the correlation coefficient value of 0.7. The networks were visualized using Cytoscape [37].

### 2.5. Molecular Evolution Analysis

The cds of each *Hsp* family was aligned by Muscle, and MEGA7 [38] was used to generate ML trees to determine their phylogeny. The EasyCodeML v1.21 [39] was used to perform the molecular evolution analysis, with the site model (SM), branch model (BM), and branch-site model (BSM) tests in the PAML package [40]. Firstly, the ratio of non-synonymous to synonymous substitution (dN/dS, ω) and the likelihood ratio tests (LRTs) were employed to evaluate the selective pressures in each *LmHsp* family. Six SMs were used to test the positive selection in each codon: M0 assumes to have the same ω for all codons, M3 assumes the ω of all codons showing a simple discrete division trend; M1a assumes only conservative sites (0 < ω < 1) and neutral sites (ω = 1) for all codons, while M2a is considered to increase the existence of positive sites (ω > 1) for all codons on the basis of M1a; the ω of all codons in M7 are assumed to belong to the matrix (0, 1) with a beta distribution, while M8 adds another type of ω (ω > 1) on the basis of M7. LRTs were used to judge whether the paired models (M0 vs. M3; M1a vs. M2a; M7 vs. M8) are significantly different [40], and to estimate whether there are positive selected sites (PSSs) (M2a vs. M1a and M8 vs. M7) under the premise of significant *p* value (*p* < 0.05).

Furthermore, BM were conducted to test the selective pressure of each *LmHsp* family. Phylogeny was constructed with all *LmHsp* cds, and each *Hsp* family was marked as the foreground branch (ω_1_), respectively, to explore the evolution rate compared with the remaining *LmHsp* families (background branch, ω_0_). BSM were conducted to test the selective pressure of single *LmHsp* gene (foreground branch, ω_1_) with 10 fish species (background branch, ω_0_), including spotted sea bass (*L. maculatus*), zebrafish (*D. rerio*), channel catfish (*I. punetaus*), stickleback (*G. aculeatus*), medaka (*O. latipes*), tilapia (*O. niloticus*), turbot (*S. maximus*), fugu (*T. rubripes*), Asian sea bass (*L. calcarifer*), and spotted gar (*L. oculatus*). With positive selection suggested (*p* < 0.05), the PSSs were further verified with high BEB posterior probabilities (>0.95). The PSSs were then retrieved from the aa sequences within functional Hsp domains from the Pfam database. 3D Structure prediction analysis was performed via Phyre2 online tool (https://www.sbg.bio.ic.ac.uk/phyre2/ accessed on 29 December 2021) [41] and modified by PyMol software (https://pymol.org/2/ accessed on 30 December 2021).

## 3. Results and Discussion

### 3.1. Genomic Landscape, Functional Domain, and Phylogeny of Hsp Superfamily in L. maculatus

**Figure 1:**
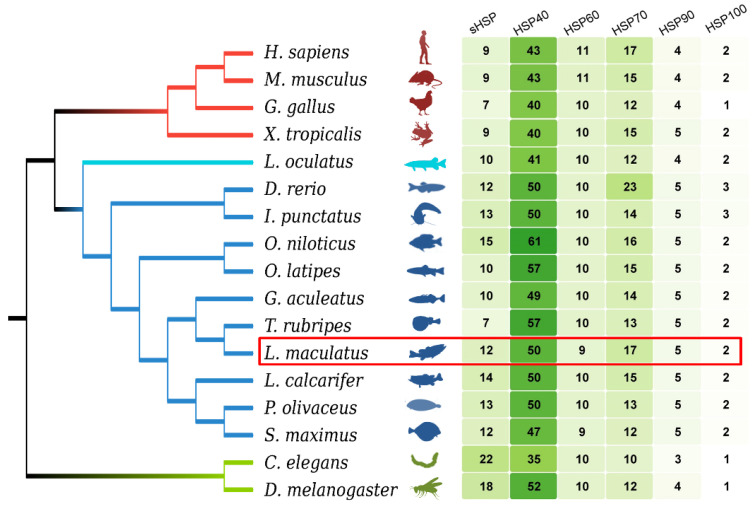
Genome-wide identification of *Hsp* families in 10 teleost species and other metazoan species, with *L. maculatus* in the red frame. Branches with different colors represent different metazoan groups. The gene numbers are illustrated with the heatmap.

Through genome-wide screen, 95 *Hsp* genes (*LmHsp*s) were identified in the *L. maculatus* genome, including 12 *sHsp*s (*LmHspb*), 50 *Hsp40*s (*LmDnaj*), 9 *Hsp10/60*s (*LmHspe*, *LmHspd*, *Lmcct*), 17 *Hsp70*s (*LmHspa*), 5 *Hsp90*s (*LmHspc*), and 2 *Hsp100*s (*LmXlp*) (Figure 1 and Table S1). The general features of *LmHsp*s were listed in Table S2, with their length ranging from 56 to 2220 amino acids (aa), and exon numbers between 1 and 53. The copy number of each *LmHsp* family was generally conserved among the selected teleost and tetrapod species (Figure 1 and Table S1), some of which (*sHsp, Hsp40* and *Hsp90*) were more in teleosts than that in the tetrapod lineages, most likely due to the teleost specific genome duplication (TSGD) event in the teleost lineages [42].

**Figure 2:**
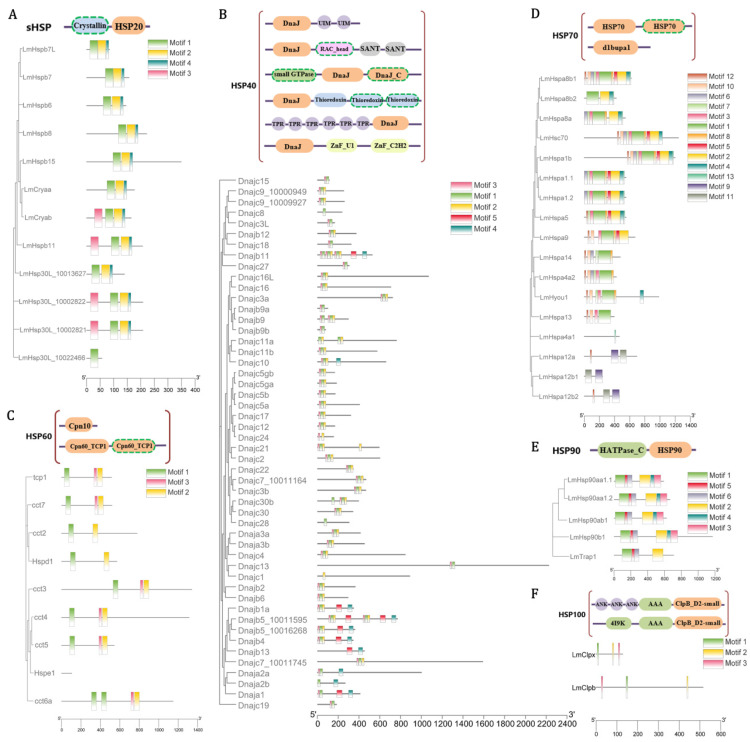
Conserved domain architecture of *LmHsp* families, as (**A**) *LmsHsp*s, (**B**) *LmHsp40*s, (**C**) *LmHsp10/60*s, (**D**) *LmHsp70*s, (**E**) *LmHsp90*s, and (**F**) *LmHsp100*s, showing along their phylogenetic relationships. The horizontal grey bars represent amino acid sequences without predicted functional domains, whereas the colored boxes represent the regions with successfully predicted motifs. The orange boxes represent the corresponding HSP domain of each family, the green boxes represent the ATPase domains, and the other boxes represent specifically annotated domains. The domains that appear only in some family members are marked with dashed frames.

Specifically, 12 *sHsp* genes were identified in the *L. maculatus* genome, which were clustered into 8 sub-families, but absent in other 4 sub-families as *Hspb1, Hspb2, Hspb3,* and *Hspb9* (Figure S2A). The *Hsp30*s was not identified in the tetrapods, but was only present in teleosts with independent duplication. In addition, as the ubiquitous family of chaperones, the structure of sHsps can be defined into three domains [43]: a conserved α-crystallin domain (ACD) with N-terminal region (NTR) and C-terminal region (CTR) on either side [44]. Most *LmsHsp*s comprised three conserved motifs (motif 1/2/4, Figure 2A) except *Hsp30L_10022466*, while *Cryab, Hspb11, Hsp30L_10002822,* and *Hsp30L_10002821* contained one extra motif 3, suggesting strong structure conservation among *LmsHsp* genes (Figure 2A).

A total of 50 *Hsp40* genes (*Dnaj*s) were identified in the *L. maculatus* genome, which were clustered into 42 sub-families (Figure S2B). Most *LmHsp40*s comprised 3 conserved motifs (motifs 1/2/3, Figure 2B) as the major DnaJ domain, while 10 *LmHsp40*s had extra motifs 4 or 5 in their sequences. Moreover, one *Hsp10* (*Hspe1*) and eight *Hsp60*s (*Hspd1* and 7 *Cct*s) were identified, with *Cct8* missing in the *L. maculatus* genome. The phylogeny supported the evolutionary relationship of *LmHspd1* and *LmHspe1*, respectively, clustered with orthologous genes of other teleost species (Figure 2C and Figure S2C). There were three conserved motifs identified among *LmHsp60* members as the Cpn60_TCP1 domain, while none were found in *LmHsp10* (Figure 2C).

Seventeen *LmHsp70* genes were identified in the *L. maculatus* genome, which contained more duplicated copies, mainly from the *Hspa1*, *Hspa8*, and *Hspa12* sub-families (Table S1 and Figure 2D). Almost all the *Hsp70* sub-families were found between human and *L. maculatus* except for *Hspa2*, *Hspa6*, *Hspa7*, and *Hsph1*, which exist in human [45] but not in fish species apart from spotted gar, zebrafish, and stickleback (Figure S2D). Thirteen conserved motifs were identified in *LmHsp70*s, and genes with closer phylogenetic relationship had similar conserved motifs. For example, motifs 9 and 11 were only annotated in *Hspa12* sub-family (Figure 2D and Figure S2D), which directly confirmed the fact that this sub-family was distantly related to other *Hsp70* genes in vertebrates [45]. Another noteworthy gene was *Hsp4a1*, which only contained one specific motif 13, and was significantly different from other *LmHsp70*s, such as *Hspa4a2*. The vertebrate *Hsp70*s were mainly scattered into nine clads, among which eight sub-families were easily distinguished, except for the sub-families *Hspa1-2-6-7-8* and *Hsc70* (Heat shock cognate 71 kDa), all clustering together (Figure S2D). This was in accordance with the fact that *Hspa1-2-6-7-8* and *Hsc70* shared a common domain called HSP1_2_6_8Nucleoid Binding Domain [46] with very similar conserved motifs (Figure S2D).

In addition, a total of five *Hsp90*s and two *Hsp100*s were collected from the *L. maculatus* genome, which was similar to that in other teleosts. The vertebrate *Hsp90* genes can be classified into four sub-families, supporting the four clads (*Hsp90aa1*, *Hsp90ab1*, *Hsp90b1* and *Trap1*) in their phylogeny (Figure S2E). Teleost *Hsp90aa1* genes were clustered into two sub-clads, *Hsp90aa1.1* and *Hsp90aa1.2*, further supporting that they were originated from the TSGD in the teleost lineage [42] (Figure S2E). Moreover, all the *LmHsp90* members contain multi-introns, and their detected six motifs are conserved with each other (Figure 2E). In total, all this information revealed the generally conserved sequence and function in teleost *Hsp* families.

### 3.2. Diverse Expression of LmHsp Genes among L. maculatus Normal Tissues

**Figure 3:**
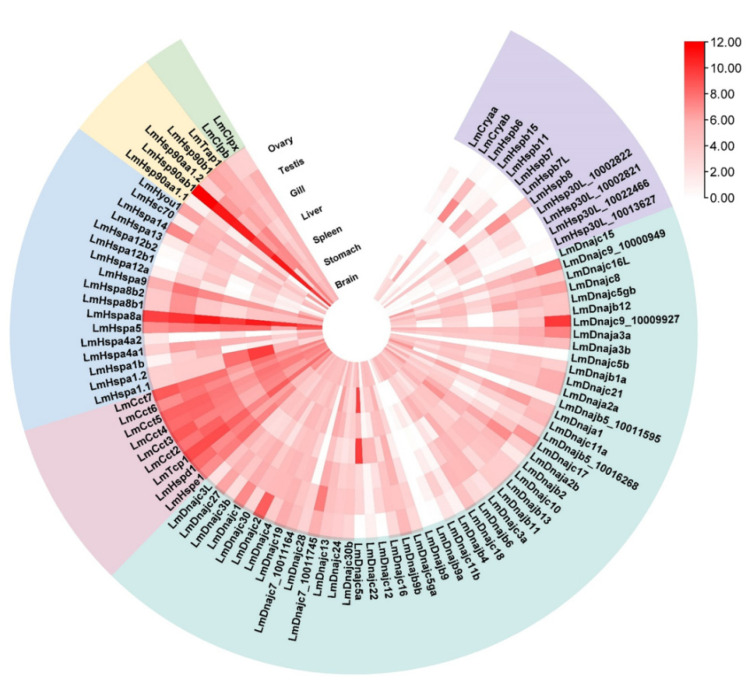
Expression profile of *LmHsp* genes among adult tissues of *L. maculatus*. The log_2_ (FPKM + 1) values are represented as 0–12 according to the colored scale bar, and the heatmap is ranked with each *LmHsp* families.

Gene expression profiling facilitates the understanding of the function and evolution of *Hsp* families. Here, we conducted transcriptome analysis with RNA-seq data from seven *L. maculatus* adult tissues (brain, stomach, liver, spleen, gill, ovary, and testis), which illustrated diverse expression profiles of the 95 *LmHsp* genes across tissues. In particular, *LmHsp10/60*s were mostly highly expressed among tissues, while *LmsHsp*s were lowly expressed (Figure 3). Interestingly, *Hspa8a* and *Hsp90ab1* were the most abundantly expressed across all tissues, suggesting their essential role in maintaining the normal physiological homeostasis in diverse tissues, whereas *Dnajb9, Dnajc5b,* and *Hspb7L* were not expressed in any tissues (Figure 3). Moreover, some *Hsp* genes showed biased expression in certain tissues. For example, *Cryab* was only expressed in brain, *Hspb11* was highly expressed in gill, and both spleen and liver had higher *Hspa1.1* and *Hspa1.2* expression than other tissues (Figure 3), which may indicate the tissue specific function of these *Hsp* members as chaperons. In addition, almost all *sHsp* genes (except *Hspb8*) were weakly expressed or even not expressed in ovary, while *Cryaa, Hspb6, Hspb7,* and *Hsp30L* were highly expressed in testis (Figure 3).

### 3.3. Regulated Expression of LmHsp Genes in Response to Salinity Change and Alkalinity Stress

**Figure 4:**
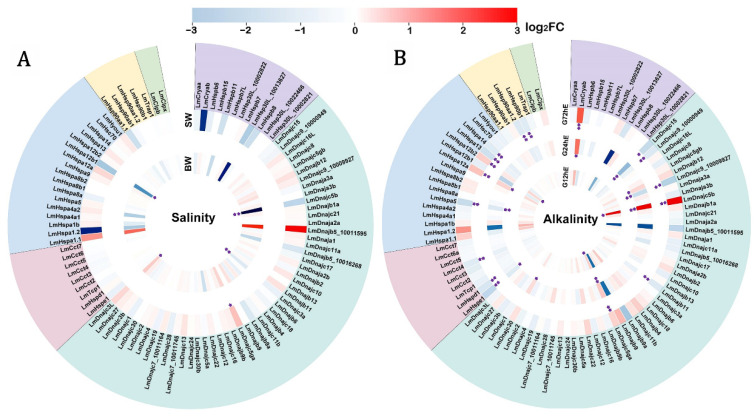
Regulated expression of *LmHsp* genes in gills of *L. maculatus* in response to (**A**) salinity change and (**B**) alkalinity stress. The heatmap was based on the log_2_FC values and ranked with each *LmHsp* family. BW and SW represent brackish and sew water groups compared to the freshwater group, while G12hE, G24hE, and G72hE indicate the alkalinity experiment duration for 12 h, 24 h, and 72 h, compared to the blank control 0 h, respectively. * indicates |log_2_FC| > 0.7 with *p* < 0.05, ** indicates |log_2_FC| > 0.7 with both *p* value and false discovery rate (FDR) < 0.05.

To investigate the functional response of *LmHsp*s to environmental changes, the regulated expression of 95 *LmHsp* genes was characterized under salinity or alkalinity stress in gills where the ion secretion and uptake happen. As chaperons, Hsps may help to maintain the homeostasis by interacting with the osmotic stress denatured proteins in gill cells. Firstly, *L. maculatus* could be well adapted to wide salinity ranges, therefore, with freshwater acclimation, the expression of *LmHsp* genes may be weakly regulated (Figure 4A and Table S3). For example, only *Hspa12b1* and three *Hsp40*s (*Dnajc3L, Dnajc3a,* and *Dnajc5b*) were significantly influenced in the brackish water group, and only *Dnajc5ga* was up-regulated in the sea water group (Figure 4A), which suggested that the gill tissues were already adapted to the stimulation caused by freshwater acclimation. Moreover, even though not significantly regulated, the expression of almost all *sHsp* members (except *Hspb7*) were reduced, and the expression of most *Hsp70*s were increased, whereas most *Hsp90* members (*Hsp90ab1*, *Hsp90b1*, and *Trap1*) did not respond to the stimulation (Figure 4A), suggesting possible functional diversification among the *LmHsp* families.

The alkalinity challenge revealed that *LmHsp*s in gills were sensitive to alkalinity stress within a short period of time (Figure 4B and Table S3), which was most likely in accordance with the good adaptation of *L. maculatus* to diverse salinities rather than alkalinity. For example, under alkalinity stress, the number of significantly regulated *LmHsp* genes (|log_2_FC| > 0.7 and *p* < 0.05) steadily increased along the test time points (Figure 4B). In particular, only 3 *LmHsp*s (*Hspa12b2*, *Dnajc5ga,* and *Dnajc5b*) were up-regulated at 12 h exposure, and 10 *LmHsp*s (*Cryab, Hspe1, Dnajb12, Dnajc5b, Dnajc5ga, Dnajc9_10009927, Dnajc15, Dnajc30, Hspa5,* and *Hspa12a*) were significantly influenced at 24 h, while at 72 h, 17 *LmHsp* genes (*Cryab, Hspe1, Hspd1, Cct4, Dnajb11, Dnajc5b, Dnajc5ga, Dnajc9_10000949, Dnajc9_10009927, Dnajc15, Hspa5, Hspa12a, Hspa12b2, Hspa13, Hspa14, Hsp90aa1.2,* and *Hsp90b1*) were significantly regulated (Figure 4B). Of these regulated *LmHsp*s, 12 were down-regulated (*Hspe1, Hspd1, Cct4, Dnajb11, Dnajc9_10000949, Dnajc9_10009927, Dnajc15, Hspa5, Hspa13, Hspa14, Hsp90aa1.2,* and *Hsp90b1*) with log_2_FC ranging from −0.72 to −2.59, whereas 7 were up-regulated (*Cryab, Dnajb12, Dnajc5b, Dnajc5ga, Dnajc30, Hspa12a,* and *Hspa12b2*) with log_2_FC ranging from 0.80 to 12.04 (Table S3), which was mostly in the *LmHsp40*, *LmHsp70,* and *LmHsp90* families but less in *LmsHsp* and *LmHsp10/60* families. *Hsp70* and *Hsp90* are the two highly conserved ATP-dependent molecular chaperones that fold and remodel proteins in almost every cellular process with co-chaperon *Dnaj*s [8,9]. The regulation of *LmHsp40*s (*Dnaj*s), *LmHsp70*s, and *LmHsp90*s indicated that they could play more essential roles in response to alkalinity stress than other *LmHsp*s. Under alkalinity stress, the ionic balance disruption occurred due to the hyperosmotic environment with protein damage [47], which can increase the levels of some *LmHsp40*s and *LmHsp70*s (Figure 4B). However, there were more down-regulated *LmHsp*s rather than up-regulated ones, possibly due to the certain degree of adaptation to alkalinity stress in *L. maculatus,* which could survive in alkaline water for more than two weeks to two months [27]. Several studies also reported down-regulation of *Hsp*s in marine organisms under diverse stimuli, such as scallops *Chlamys farreri* and *Patinopecten yessoensis Hsp70B2* genes in response to toxic dinoflagellates [16,17], ascidian *Ciona savignyi Hsp20/60/70/90* genes under both low and high salinity stresses [48], and European seabass *Dicentrarchus labrax Hsp70* in low salinity conditions [47]. Further investigation is warranted with a possible delayed response in *LmHsp* expression level as well as the regulatory heat shock factor (Hsf) of these *LmHsp*s. In addition, the expression of *Hspa8a* and *Hsp90ab1* did not exhibit differential regulation (Figure 4B), although they showed high abundance in both normal and challenged gill tissues (Figure 3 and Table S3), indicating that these genes did not participate in the response to alkalinity stress but may be needed to maintain the physiological homeostasis.

### 3.4. Coordinated Regulation of LmHsp Co-Chaperones under Alkalinity Stress

**Figure 5:**
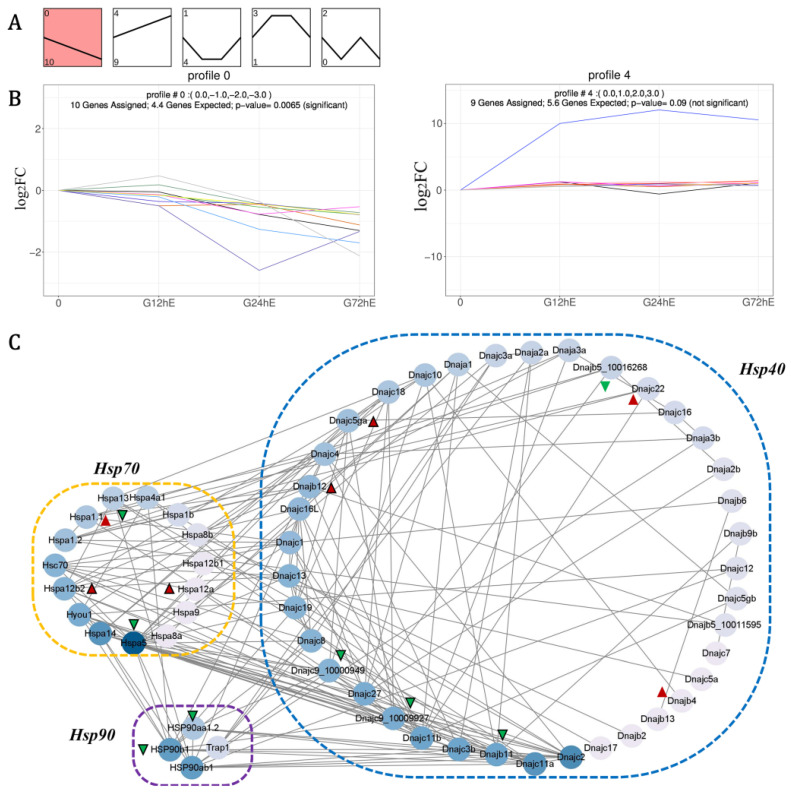
The expression trend and coordinated regulation of *LmHsp40-70-90* co-chaperons in response to alkalinity stress. (**A**) Expression patterns enriched in five profiles with the number of *LmHsp*s indicated; (**B**) Expression trends of *LmHsp* genes enriched in the down-regulated profile 0 and up-regulated profile 4; (**C**) Protein-protein interaction (PPI) according to the PCC of *LmHsp* expression under alkalinity stress. Green and red triangles represent *Hsp*s from profiles 0 and 4, respectively, and triangles with black frame are the significantly regulated *LmHsp*s in response to alkalinity stress.

When the *Hsp*s repair misfolded proteins in a simplified model, *Hsp70* initially facilitates the re-folding of hydrophobic residue stretches into their native state together with *Hsp40*s (*Dnaj*s), whereas *Hsp90* catalyzes a step later in protein folding and activation [8,11]. With the possible co-chaperon among *Hsp40*s, *Hsp70*s, and *Hsp90*s, to further excavate their associated regulation, the expression trend and Pearson’s correlation coefficient (PCC) of *LmHsp40-70-90* genes under alkalinity stress were further investigated. As a result, 24 *LmHsp40-70-90* genes were enriched into 4 expression profiles (Figure 5A and Table S4), among which 10 *LmHsp*s were enriched in profile 0 (declined expression) and 9 *LmHsp*s were enriched in profile 4 (raised expression) (Figure 5B). In profile 0, the expression of two *LmHsp70*s (*LmHspa5* and *LmHspa13*), two *LmHsp90*s (*LmHsp90aa1.2* and *LmHsp90b1*), and six *LmHsp40*s (*LmDnajc15, LmDnajc9_10000949, LmDnajc9_10009927, LmDnajb5_10016268, LmDnajb11,* and *LmDnajb9a*) were continuously down-regulated (Figure 5B), while in profile 4, the expression of three *LmHsp70*s (*LmHspa1.1, LmHspa12a,* and *LmHspa12b2*) and six *LmHsp40*s (*LmDnajb12, LmDnajc5b, LmDnajb4, LmDnajc5ga, LmDnajc22,* and *LmDnajc30*) were continuously up-regulated (Figure 5B). These transcriptional profiles may be suggestive of coordinated regulation of the *Hsp40-70-90* genes and could thus comprise the possible component of alkalinity adaptation in *L. maculatus*, which warrants further verification.

In addition, according to PCC > 0.7, 15 *LmHsp40-70-90* members from profile 0 (8 *Hsp*s) and profile 4 (7 *Hsp*s) may be coordinately regulated with each other (Figure 5C and Table S5). For example, the up-regulated *LmHspa12b2* was positively correlated with *LmDnajb12* and negatively correlated with *LmDnajc9*, while the down-regulated *LmHsp90b1* and *LmHsp90aa1.2* were positively correlated with *LmDnajc9* or *LmDnajb11* (Figure 5C and Table S5), which may indicate *LmDnajc9* as the key co-chaperon of *LmHspa12b2*, *LmHsp90b1,* and *LmHsp90aa1.2*. Moreover, the down-regulated *LmHspa5* also co-expressed with *LmDnajb11, LmDnajc9,* and *LmHsp90b1*, while negatively related to *LmHspa12b2* and *LmDnajc5ga*. These protein-protein interaction correlations among Hsp40-70-90 members could also be observed in model organisms such as zebrafish and human (Figure S3), indicating functional conservation of these Hsp co-chaperons. Moreover, the expression trends and PCC relationship also revealed that the down-regulated co-expression of *LmHsp40-70-90* members may reflect their corporation, whereas the up-regulation of *LmHsp40*s and atypical *LmHspa12*s may supplement the loss caused by down-regulation (Figure 5C).

### 3.5. Evolutionary Dynamics of LmHsp Families in L. maculatus

**Figure 6:**
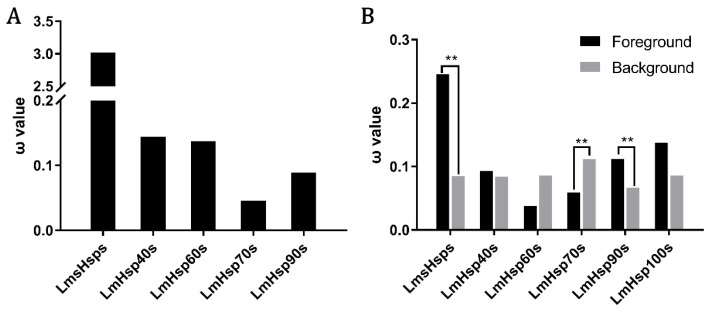
Molecular evolution of *LmHsp* genes in *L. maculatus*. (**A**) ω values of each *LmHsp* family from site model tests; (**B**) Branch-model tests of each *LmHsp* family from *L. maculatus*, black and gray columns indicate ω values of each *LmHsp* family (ω_1_, foreground branch) and other *LmHsp* families (ω_0_, background branch), respectively. ** indicates *p* < 0.01.

To gain insights of whether the regulated *LmHsp*s may function under adaptive evolution in dynamic aquatic environments, the molecular evolution of *LmHsp* families was evaluated through a series of model tests, including site model (SM), branch model (BM), and branch-site model (BSM) via PAML. Firstly, the ω values (dN/dS) of each *LmHsp* family were obtained via the SM tests, which revealed that the evolutionary rate (ω) of the *LmsHsp* family was generally higher than that of other *LmHsp* families (Figure 6A and Table S6). For example, the *LmsHsp* family exhibited rapid evolution with ω > 1, while the *LmHsp70* and *LmHsp90* families were more conserved during evolution (ω < 0.1) (Figure 6A). In addition, with BM tests for the 95 *LmHsp* genes labeling each family as the foreground branch (ω_1_), respectively, it confirmed that in *L. maculatus*, *sHsp*s represented faster molecular evolution rates than other *Hsp* families (*p* < 0.01), whereas *LmHsp70*s had the opposite evolutionary pattern with high sequence conservation (Figure 6B and Table S6). This was in line with the results obtained in the SM tests (Figure 6A). Moreover, *LmHsp90*s represented rapid evolution in the BM test but not in the SM test. These results suggested that although most *Hsp* genes were deemed to be highly conserved among teleost species, the molecular evolutionary rates of *sHsp*s and *Hsp90*s were generally faster in *L. maculatus*.

Accordingly, the BSM tests of *LmsHsp*s, *LmHsp70*s and *LmHsp90*s, which represented significant altered evolutionary rates in *L. maculatus* (Figure 6B), were further conducted against *Hsp*s of other teleost lineages, and the putative positively selected sites (PSSs) were detected in 10 genes (*p* < 0.01, BEB probability > 0.95), including *Hspa4a1, Hspa4a2, Hspa8a, Hspa9, Hspa12b1, Hspa12b2, Hsc70, Hsp90aa1.2, Hsp90ab1,* and *Hsp90b1* (Table S7). The genes with PSS detected were mainly found in seven *Hsp70* and three *Hsp90* members but not in *sHsp*s (Table S7), among which *Hspa12b2, Hsp90aa1.2,* and *Hsp90b1* were also significantly regulated in response to alkalinity stress (Figure 5C). Therefore, in an adverse aquatic environment, the *sHsp* family may be under stronger evolutionary pressure in both *L. maculatus* and other teleosts (Figure 6), whereas in *L. maculatus*, some members of *Hsp70* and *Hsp90* families may evolve more rapidly than other teleosts (Table S7), possibly demonstrating the good adaptation of *L. maculatus* to the environmental changes, such as water temperature, salinity, and alkalinity.

**Figure 7:**
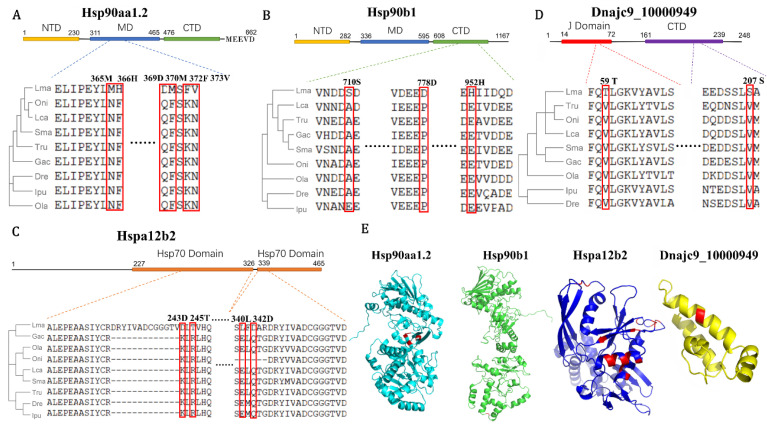
Examples of positively selected sites (PSSs) in (**A**) *Hsp90aa1.2*, (**B**) *Hsp90b1*, (**C**) *Hspa12b2,* and (**D**) *Dnajc9* functional domains from branch-site model tests. The partial aa sequence alignments of the selected vertebrates with their phylogeny are presented. Red frames indicate PSSs detected with their location at the bar above. Omitted multiple sequence alignment between the two segments are represented by dots. NTD, N-terminal domain; MD, middle domain; and CTD, C-terminal domain. (**E**) Schematic diagram of 3D structure of the above-mentioned LmHsp members with PSSs labeled in red. Only 710 S was predicted in the Hsp90b1 3D structure.

In addition, with BSM tests of the 15 putative correlated *Hsp40-70-90* co-chaperons in response to alkalinity stress (Table 1 and Figure 5C), the PSSs in the four significantly regulated *Hsp*s, *Hsp90aa1.2, Hsp90b1, Hspa12b2,* and *Dnajc9*, were detected in their conserved functional domains (Figure 7). Hsp90 chaperones normally exist as a dimer, and each part contains three domains: the N-terminal domain (NTD) with ATP hydrolytic activity, the middle domain (MD) to interact with Hsp70, and the C-terminal domain (CTD) to participate in identification and interaction of clients [49]. The eukaryotic Hsp90 proteins also contain a C-terminal extension of the MEEVD motif to help them bind to co-chaperones [8,49]. The fast-evolved PSSs of *Hsp90aa1.2* and *Hsp90b1* were mainly present in the MD and CTD (Figure 7A,B,E), which could most likely affect their interaction efficiency with Hsp70 proteins. Moreover, gene duplication is one of the most essential ways to develop functional divergence through environmental adaptation [42]. Both Hsp90 and Hsp70 function in an ATP-dependent scenario and require the involvement of a protein-named activator of Hsp90 ATPase homolog (Hsp90aa1), which interacts with Hsp90 and Hsp70 to stimulate ATPase activity [8,11]. Two putative *Hsp90aa1* genes were identified in *L. maculatus* due to TSGD, while only one (*LmHsp90aa1.2*) was significantly induced in response to alkalinity (Figure 4B). Therefore, there can be functional diversification between the duplicated *Hsp90aa1* genes in *L. maculatus*, with *Hsp90aa1.2* both evolving significantly fast and regulated under alkalinity stress, but not in *Hsp90aa1.1* (Figure 4 and Table 1).

Moreover, Hsp70s are regarded as the most well-known member of the Hsp superfamily, which can act as co-chaperones of Hsp40s and Hsp90s involved in the folding and remodeling of various proteins [9]. Generally, the typical Hsp70 members consist of two regions, a conserved nucleotide-binding domain (NBD) with an ATP binding site, and a substrate-binding domain (SBD), which can mediate substrate or co-chaperone binding [50]. Those lacking NBD or SBD, such as Hspa4 and Hspa12 (Figure 2D), are considered atypical members of the Hsp70 family [45]. Here, only the atypical *Hspa12* genes in *L. maculatus* were up-regulated under alkalinity stress, while other typical *Hsp70*s (*LmHspa5*, *LmHspa13,* and *LmHspa14*) were mostly down-regulated together with their co-chaperon *Hsp90*s, indicating possible functional diversification. There are two Hsp70 domains detected in LmHspa12b2, and the PSSs mainly exist in its C-terminal domain (Figure 7C,E), indicating its possible binding specificity alternation. Moreover, PSSs were also detected in the J domain and CTD of LmDnajc9, which is essential in stimulating Hsp70 ATPase activity (Figure 7D,E) and also as the putative key co-chaperon with LmHspa12b2 and LmHsp90aa1.2 (Figure 5C). Therefore, these fast evolved loci in functional domains may contribute to the *Hsp*s functional co-evolution in *L. maculatus* (Figure 7E), and it may also suggest their vital correlated roles in response to diverse environmental stresses in *L. maculatus* through adaptive evolution. Further investigation is warranted among these Hsp40-70-90 co-chaperons to better understand their function and adaptive evolution in *L. maculatus*.

## 4. Conclusions

*Hsp*s contribute to mediate stress responses by refolding damaged proteins to prevent their aggregation. This study provided a systematic genomic and transcriptomic survey of 95 *Hsp* genes in spotted sea bass (*L. maculatus*), which has good adaptability in saline-alkaline waters. Diverse expression regulation of *LmHsp*s was observed in responses to alkalinity stress, which was majorly responded in coordinated *LmHsp40-70-90* families through the adaptive recruitment of these co-chaperons. These findings are useful for understanding the diverse functions of *Hsp* co-chaperon network and adaptive evolution in teleost species. Further investigation on the functions of teleost *Hsp* network will provide a more detailed explanation about their adaptation mechanisms against different environmental challenges and warrant a better feasibility regarding the cultivation of *L. maculatus* in alkaline water.

## Figures and Tables

**Table 1:** Branch-site model tests for the 15 coordinated regulated *LmHsp40-70-90* co-chaperons (Figure 5C) between *L. maculatus* and other teleost species.

Gene ID	*p* Value	Positive Selected Sites
*Hspa1.1*	1.0000	/
*Hspa5*	1.0000	*/*
*Hspa12a*	0.0534	8 S 0.919
***Hspa12b2***	**0.0000**	32 P 0.958 *, 36 T 0.918, 39 V 0.935, 41 L 0.907, 43 G 0.923, 46 P 0.936, 49 R 0.906, 112 C 0.903, 321 D 0.995 **, 323 T 1.000 **, 327 I 0.907, 339 K 0.999 **, 340 A 0.998 **, 341 S 0.929, 343 E 0.999 **, 344 L 0.963 *, 346 A 1.000 **, 347 K 1.000 **, 351 R 0.998 **, 353 V 0.999 **, 355 F 0.997 **, 366 P 0.999 **, 367 M 0.997 **, 368 L 0.998 **, 370 K 0.999 **, 371 A 0.998 **, 372 V 0.963 *, 374 K 1.000 **, 375 A 0.999 **, 377 G 0.999 **, 379 T 1.000 **, 384 I 0.963 *, 408 S 1.000 **, 409 Q 1.000 **, 411 H 0.997 **, 418 L 1.000 **, 419 F 0.866, 420 D 0.997 **
*Hspa13*	**0.0039**	/
***Hsp90aa1.2***	**0.0000**	16 G 0.908, 22 D 0.916, 431 M 0.978 *, 432 H 0.996 **, 488 D 0.974 *, 489 M 0.982 *, 491 F 1.000 **, 492 V 0.974 *
***Hsp90b1***	**0.0000**	723 S 0.983 *, 791 D 0.998 **, 965 H 0.981 *, 1099 E 0.940
*Dnajb4*	0.6613	239 N 0.861
*Dnajb5_10016268*	1.0000	289 Y 0.507
*Dnajb11*	1.0000	/
*Dnajb12*	1.0000	/
*Dnajc5ga*	1.0000	/
***Dnajc9_10000949***	**0.0079**	77 R 0.825, 95 K 0.961 *, 96 E 0.990 *, 97 A 0.522, 109 V 0.978 *, 130 V 0.980 *, 141 K 0.930, 204 V 0.921, 205 Q 0.896, 206 H 0.944, 207 Q 0.791, 211 D 0.921, 216 S 0.596, 220 C 0.970*, 244 F 0.710, 269 A 0.614, 276 M 0.650, 283 D 0.911, 286 V 0.972 *, 318 S 0.661, 320 D 0.976 *, 338 N 0.994 **, 350 E 0.700
*Dnajc9_10009927*	1.0000	/
*Dnajc22*	1.0000	/

The ancestral branch leading to *L. maculatus* was set as the foreground branch (ω_1_). Sites with the BEB posterior probabilities higher than 90% were presented, with those higher than 95% marked with * and higher than 99% marked with ** and in bold. *p* values < 0.05 were in bold.

## Data Availability

The transcriptome datasets used in this study can be found in the NCBI Sequence Read Archive (SRA) BioProject PRJNA611641 and PRJNA515986, as well as *L. maculatus* tissue transcriptomes, including brain-SRR7528887, stomach-SRR7528884, spleen-SRR7528888, liver-SRR7528886, gill-SRR7528883, testis-SRR7528885 and ovary-SRR2937376.

## Supplementary Materials

The following supporting information can be downloaded at: https://www.mdpi.com/article/10.3390/biology11030353/s1, Table S1: Detailed copy number of *Hsp* family genes in selected vertebrate genomes; Table S2: Summarized information of *LmHsp* genes; Table S3: Differential expression of *LmHsp*s under salinity change and alkalinity stress by edgeR analysis; Table S4: Trend analysis for the *LmHsp40-70-90* gene expression under alkalinity stress; Table S5: Protein-protein interaction (PPI) network for the *LmHsp40-70-90* gene expression under alkalinity stress; Table S6: Molecular evolution analysis of the *LmHsp* genes among teleost lineages via PAML; Table S7: Branch-site model tests of the *LmHsp* genes with other teleost species via PAML; Figure S1: Principal component analysis (PCA) with FPKM values of all transcriptome samples under salinity change and alkalinity stress; Figure S2: Structure, motif and phylogeny of *LmHsp* families, showing phylogenetic relationship along with their conserved motifs. (**A**) *LmsHsp*s, (**B**) *LmHsp40*s, (**C**) *LmHsp10/60*s, (**D**) *LmHsp70*s, (**E**) *LmHsp90*s, and (**F**) *LmHsp100*s. Abbreviations: *Hsa: H. sapiens; Mmu: M. musculus; Gga: G. Gallus; Xtr: X. tropicalis; Loc: L. oculatus; Dre: D. rerio; Ipu: I. punctatus; Ola: O. latipes; Gac: G. aculeatus; Sma: S. maximus; Tru: T. rubripes; Oni: O. niloticus; Lca: L. calcarifer; Lma: L. maculatus*; Figure S3: Protein-protein interaction (PPI) of *Hsp*s from zebrafish and human.
