# Supplementary material for: Genomic and Transcriptomic Landscape and Evolutionary Dynamics of Heat Shock Proteins in Spotted Sea Bass (Lateolabrax maculatus) under Salinity Change and Alkalinity Stress
Source: Biology (Basel). 2022 Feb 23;11(3):353. doi: 10.3390/biology11030353 (PMC8945262; doi:10.3390/biology11030353)
Supplement: Supplementary file 1 [file biology-11-00353-s001.zip › Table S1. Copy number.pdf]

Table S1. Detailed copy number of *Hsp* genes in selected vertebrate genomes.

Name	*Hsa*	*Mmu*	*Gga*	*Xtr*	*Loc*	*Dre*	*Sma*	*Oni*	*Gac*	*Ola*	*Tru*	*Ipu*	*Lca*	*Lma*
Hsp90aa1	1	1	1	2	1	2	2	2	2	2	2	2	2	2
Hsp90ab1	1	1	1	1	1	1	1	1	1	1	1	1	1	1
Hsp90b1	1	1	1	1	1	1	1	1	1	1	1	1	1	1
Trap1	1	1	1	1	1	1	1	1	1	1	1	1	1	1
**Tot *Hsp90***	**4**	**4**	**4**	**5**	**4**	**5**	**5**	**5**	**5**	**5**	**5**	**5**	**5**	**5**
Hspe1	1	1	1	1	1	1	1	1	1	1	1	1	1	1
Hspd1	1	1	1	1	1	1	1	1	1	1	1	1	1	1
Tcp1	1	1	1	1	1	1	1	1	1	1	1	1	1	1
Cct2	1	1	1	1	1	1	/	1	1	1	1	1	1	1
Cct3	1	1	1	1	1	1	1	1	1	1	1	1	1	1
Cct4	1	1	1	1	1	1	1	1	1	1	1	1	1	1
Cct5	1	1	1	1	1	1	1	1	1	1	1	1	1	1
Cct6	2	2	1	1	1	1	1	1	1	1	1	1	1	1
Cct7	1	1	1	1	1	1	1	1	1	1	1	1	1	1
Cct8	1	1	1	1	1	1	1	1	1	1	1	1	1	/
**Tot Hsp60**	**11**	**11**	**10**	**10**	**10**	**10**	**9**	**10**	**10**	**10**	**10**	**10**	**10**	**9**
Hspa1	3	3	/	3	1	2	/	1	1	1	1	/	1	3
Hspa2	1	1	1	1	/	/	/	/	1	/	/	/	1	
Hspa4	2	2	2	1	2	3	3	3	3	3	3	3	3	2
Hspa5	1	1	1	1	1	1	1	2	1	1	1	1	1	1
Hspa6	1	/	/	/	/	/	/	/	/	/	/	/	/	/
Hspa7	1	/	/	/	/	/	/	/	/	/	/	/	/	/
Hspa8	1	1	1	1		2	1	2	2	2	1	2	2	3
Hspa9	1	1	1	1	1	1	1	1	1	1	1	1	1	1
Hspa12	2	2	2	2	2	5	2	2	2	2	2	2	2	3
Hspa13	1	1	1	1	1	1	1	1	1	1	1	1	1	1
Hspa14	1	1	1	1	1	1	1	1	1	1	1	1	1	1
Hsph1	1	1	1	1	1	1	/	/	/	/	/	/	/	/
Hyou1	1	1	1	1	1	1	1	1	1	1	1	1	1	1
Hsc70	/	/	/	/	/	1	1	1	/	1	1	1	1	1
Hsp70	/	/	/	1	1	4	/	1	/	1		1	/	/
**Tot *Hsp70***	**17**	**15**	**12**	**15**	**12**	**23**	**12**	**16**	**14**	**15**	**13**	**14**	**15**	**17**
Hspb1	1	1	1	1	1	1	1	1	1	/	1	1	1	/
Hspb2	1	1	1	1	1	1	/	/	/	/	/	1	/	/
Hspb3	1	1	1	1	1	1	/	/	/	/	/	1	/	/
Hspb6	1	1	/	1	/	1	1	1	1	1	1	1	1	1
Hspb7	1	1	1	2	2	1	3	3	2	1	1	2	3	2
Hspb8	1	1	1	1	1	1	1	1	1	1	1	1	1	1
Hspb9	1	1	/	/	/	1	1	1	1	/	/	1	1	/
Hspb11	/	/	/	/	1	1	1	1	/	1	1	1	1	1

Hspb15	/	/	/	/	1	1	/	1	/	1	/	1	1	1
Cryaa	1	1	1	1	1	1	1	1	1	1	1	1	1	1
Cryab	1	1	1	1	1	2	1	1	1	1	1	2	1	1
Hsp30	/	/	/	/	/	/	2	4	2	3	/	/	3	4
**Tot s*Hsp***	**9**	**9**	**7**	**9**	**10**	**12**	**12**	**15**	**10**	**10**	**7**	**13**	**14**	**12**
Dnaja1	1	1	1	1	1	1	1	1	/	2	1	1	1	1
Dnaja2	1	1	1	1	1	2	1	2	2	2	2	2	2	2
Dnaja3	1	1	1	1	1	2	2	2	2	2	2	2	2	2
Dnaja4	1	1	1	1	1	/	1	1	1	1	1	1	1	/
Dnajb1	1	1	1	1	1	2	2	3	1	3	3	2	2	1
Dnajb2	1	1	1	1	1	1	1	1	1	1	1	1	/	2
Dnajb3	1	1	/	/	/	/	/	/	/	/	/	/	/	/
Dnajb4	1	1	1	1	1	1	1	1	1	1	1	1	1	1
Dnajb5	1	1	1	1	1	1	2	2	1	2	3	2	1	2
Dnajb6	1	1	1	1	1	2	2	2	2	2	2	2	1	1
Dnajb7	1	1	/	/	/	/	/	/	/	/	/	/	/	/
Dnajb8	1	1	/	/	/	/	/	/	/	/	/	/	/	/
Dnajb9	1	1	1	1	1	2	1	3	2	2	2	2	2	3
Dnajb11	1	1	1	1	1	1	1	1	1	/	1	1	1	1
Dnajb12	1	1	1	1	1	2	2	2	2	2	2	2	2	1
Dnajb13	1	1	1	1	1	1	1	1	1	1	1	1	1	1
Dnajb14	1	1	1	1	1	/	/	1	1	1	1	/	1	/
Dnajc1	1	1	1	1	1	1	1	1	1	1	1	1	1	1
Dnajc2	1	1	1	1	1	1	1	1	1	1	1	1	1	1
Dnajc3	1	1	1	1	2	2	2	2	2	2	2	2	2	3
Dnajc4	1	1	1	1	/	1	1	1	1	1	1	1	1	1
Dnajc5	1	1	1	1	3	5	2	5	5	5	5	5	5	4
Dnajc6	1	1	1	1	1	1	1	1	1	1	1	1	1	/
Dnajc7	1	1	1	1	1	1	1	2	1	2	2	1	1	2
Dnajc8	1	1	1	1	1	1	1	1	1	1	1	1	1	1
Dnajc9	1	1	1	1	1	1	1	1	1	1	1	1	1	2
Dnajc10	1	1	1	1	1	1	1	1	1	1	1	1	1	1
Dnajc11	1	1	1	1	1	2	2	2	2	2	2	/	2	2
Dnajc12	1	1	1	1	1	1	1	1	1	1	1	1	1	1
Dnajc13	1	1	1	1	1	/	/	/	1	/	1	/	1	1
Dnajc14	1	1	1	1	1	1	1	1	/	1	1	1	1	/
Dnajc15	1	1	1	1	1	1	1	/	1	1	1	1	1	1
Dnajc16	1	1	1	1	1	2	2	2	2	2	2	2	2	2
Dnajc17	1	1	1	1	1	1	1	1	1	1	1	1	1	1
Dnajc18	1	1	1	1	1	1	1	1	1	1	1	1	1	1
Dnajc19	1	1	1	1	1	1	1	1	1	1	1	1	1	1
Dnajc21	1	1	1	1	1	1	1	1	1	1	1	1	1	1
Dnajc22	1	1	1	1	1	1	1	1	1	1	1	1	1	1
Dnajc24	1	1	1	1	1	1	1	3	/	1	1	1	/	1

Dnajc25	1	1	1	1	1	1	1	1	1	1	1	1	1	/
Dnajc27	1	1	1	1	1	1	1	1	1	1	1	1	1	1
Dnajc28	1	1	1	1	1	1	/	1	/	1	/	1	1	1
Dnajc30	1	1	1	1	/	1	2	2	1	2	2	1	1	2
**Tot *Hsp40***	**43**	**43**	**40**	**40**	**41**	**50**	**47**	**61**	**49**	**57**	**57**	**50**	**50**	**50**
Clpb	1	1	/	1	1	1	1	1	1	1	1	1	1	1
Clpx	1	1	1	1	1	2	1	1	1	1	1	2	1	1
**Tot *Hsp100***	**2**	**2**	**1**	**2**	**2**	**3**	**2**	**2**	**2**	**2**	**2**	**3**	**2**	**2**
**Total**	**86**	**86**	**74**	**81**	**79**	**100**	**87**	**109**	**90**	**99**	**94**	**95**	**96**	**95**
