# Supplementary material for: Genomic and Transcriptomic Landscape and Evolutionary Dynamics of Heat Shock Proteins in Spotted Sea Bass (Lateolabrax maculatus) under Salinity Change and Alkalinity Stress
Source: Biology (Basel). 2022 Feb 23;11(3):353. doi: 10.3390/biology11030353 (PMC8945262; doi:10.3390/biology11030353)
Supplement: Supplementary file 1 [file biology-11-00353-s001.zip › Table S2. Gene feature.pdf]

Table S2. Summarized information of *LmHsp* genes.

Gene ID	Size (amino acid)	HSP domain (amino acid)	Exon numbers
LmHspb6	154	52 ~ 144	3
LmHspb7	156	60 ~ 156	3
LmHspb7L	84	3 ~ 84	2
LmHspb8	221	109 ~ 195	3
LmHspb11	206	82 ~ 182	1
LmHspb15	354	88 ~ 184	4
LmCryaa	176	66 ~ 165	3
LmCryab	164	60 ~ 154	3
LmHsp30L_10002821	207	78 ~ 178	1
LmHsp30L_10002822	207	78 ~ 178	1
LmHsp30L_10013627	139	10 ~ 95	1
LmHsp30L_10022466	56	5 ~ 56	1
LmHspd1	569	47 ~ 527	10
LmHspe1	104	10 ~ 102	4
LmTcp1	514	52 ~ 495	11
LmCct2	776	30 ~ 287, 284 ~ 439	21
LmCct3	1340	490 ~ 964	32
LmCct4	1312	41 ~ 536	19
LmCct5	541	44 ~ 536	11
LmCct6a	1148	282 ~ 867	33
LmCct7	517	3 ~ 495	10
LmDnaja1	406	5~60,111~333,138~204	8
LmDnaja2a	995	7~62,144~240	13
LmDnaja2b	263	1~39,115~190	6
LmDnaja3a	409	140~198	7
LmDnaja3b	447	91~149	10
LmDnajb1a	344	3~60,168~327	3
LmDnajb2	359	2~61	9
LmDnajb4	340	3~60,164~323	3
LmDnajb5-10011595	766	108~165,258~409,439~496,589~748	8
LmDnajb5-10016268	356	3~60,179~338	3
LmDnajb6	290	2~61	8
LmDnajb9	293	25~77,102~159	3
LmDnajb9a	96	25~80	1
LmDnajb9b	74	25~74	1
LmDnajb11	523	26~78,79~137,164~222,274~467	13
LmDnajb12	368	109~166	8
LmDnajb13	447	269~428	12
LmDnajc1	883	70~99	12
LmDnajc2	596	77~143	15
LmDnajc3a	718	608~669	15

LmDnajc3b	461	353~414	11
LmDnajc3L	161	98~159	4
LmDnajc4	840	34~92	22
LmDnajc5a	400	15~73	11
LmDnajc5b	167	14~72	4
LmDnajc5ga	179	18~76	4
LmDnajc5gb	161	16~74	4
LmDnajc7-10011164	462	370~433	12
LmDnajc7-10011745	1585	378~441	40
LmDnajc8	232	54~94	8
LmDnajc9-10000949	248	14~72	6
LmDnajc9-10009927	255	14~72	5
LmDnajc10	654	36~94	20
LmDnajc11a	755	2~52,172~238	20
LmDnajc11b	569	14~75	15
LmDnajc12	163	13~71	5
LmDnajc13	2220	1274~1332	53
LmDnajc15	110	66~108	4
LmDnajc16	701	30~87	12
LmDnajc16L	1064	35~92	19
LmDnajc17	316	11~69	11
LmDnajc18	320	104~184	7
LmDnajc19	179	120~163	5
LmDnajc21	589	2~61,387~420	14
LmDnajc22	339	277~334	3
LmDnajc24	151	7~55	4
LmDnajc27	301	244~301	6
LmDnajc28	300	44~119	1
LmDnajc30	337	164~222	1
LmDnajc30b	391	215~273	1
LmHspa1.1	551	1 ~ 526	1
LmHspa1.2	551	1 ~ 526	1
LmHspa1b	1200	569 ~ 1175	17
LmHspa4a1	463	2 ~ 315	10
LmHspa4a2	424	3 ~ 392	9
LmHspa5	552	50 ~ 532	7
LmHspa8a	544	1 ~302, 326 ~ 505	6
LmHspa8b1	622	6 ~ 612	13
LmHspa8b2	423	18 ~ 388	13
LmHspa9	671	48 ~ 637	15
LmHspa12a	694	79 ~ 265, 324 ~ 437	12
LmHspa12b1	241	1 ~ 121	3
LmHspa12b2	465	227 ~ 326, 339 ~ 465	10
LmHspa13	396	33 ~ 385	5

LmHspa14	476	3 ~ 131, 180 ~ 471	11
LmHsc70	1244	438 ~ 1039	23
LmHyou1	984	28 ~ 684	22
LmHsp90aa1.1	588	192 ~ 579	9
LmHsp90aa1.2	662	238 ~ 649	10
LmHsp90ab1	622	190 ~ 604	10
LmHsp90b1	1167	257 ~ 783	26
LmTrap1	707	302 ~ 707	18
LmClpb	515	396 ~ 489	15
LmClpx	128	51 ~ 86	2
