# Supplementary material for: Genomic and Transcriptomic Landscape and Evolutionary Dynamics of Heat Shock Proteins in Spotted Sea Bass (Lateolabrax maculatus) under Salinity Change and Alkalinity Stress
Source: Biology (Basel). 2022 Feb 23;11(3):353. doi: 10.3390/biology11030353 (PMC8945262; doi:10.3390/biology11030353)
Supplement: Supplementary file 1 [file biology-11-00353-s001.zip › Table S7. BSM-PSS-new.pdf]

Table S7. Branch-site model tests of Hsp70s among teleost species. The branch leading to each *LmHsp* gene was set as the foreground branch (ω_1). Sites with the BEB posterior probabilities higher than 90% are presented, with those higher than 95% marked with * and higher than 99% marked with **. p values < 0.05 are in bold.

Gene ID	*p* value	Positive Selected Sites
Hspe1	**0.0329**	/
Hspb6	1.0000	/
Hspb7	0.9848	/
Hspb8	0.2118	/
Hspb11	0.3686	/
Hspb15	0.1229	/
Cryaa	1.0000	/
Cryab	1.0000	/
Hsp30	0.0855	/
Hspd1	**0.0000**	**577 Q 0.999**, 586 S 0.999**, 587 P 0.998**, 595 T 0.999**, 597 S 0.998**, 606 P 0.997**, 608 A 0.979*, 612 P 0.993**, 613 S 0.998**, 614 P 0.999**, 615 P 0.999****, 617 S 0.919, **618 P 0.999**, 619 S 0.995**, 621 P 0.999**, 623 T 0.995**, 624 P 0.999**, 625 P 0.999****
Hspa1.1	1.0000	/
Hspa1.2	0.5008	1213 V 0.932
Hspa1b	0.5593	/
Hspa4a1	**0.0023**	**55 S 0.991**, 755 C 0.987***
Hspa4a2	**0.0000**	**96 C 0.972*, 393 V 0.999**, 394 Q 1.000**, 435 S 1.000**, 436 R 0.999**, 442 G 0.999**, 443 P 1.000**, 445 S 1.000**, 446 E 0.999**, 449 K 0.989*, 488 A 0.965*, 498 F 0.998**, 500 F 0.999**, 501 H 0.997**, 626 A 0.997**, 628 S 0.992****
Hspa5	1.0000	/
Hspa8a	**0.0000**	**383 K 0.990**, 386 Q 1.000**, 387 T 0.992**, 388 S 1.000**, 389 V 0.996**, 390 C 1.000**, 391 A 0.994**, 393 R 0.964*, 400 T 0.998**, 403 S 0.997**, 404 N 0.997****, 407 L 0.946, **408 F 1.000**, 409 V 0.994**, 411 I 0.999****, 426 R 0.941, **427 A 1.000**, 428 T 0.996**, 429 E 1.000**, 442 Y 0.999**, 444 Q 1.000****
Hspa8b1	1.0000	/
Hspa8b2	1.0000	/
Hspa9	**0.0000**	**2 G 0.998**, 5 Q 0.967*, 10 H 0.999****, 12 G 0.905, **30 G 0.999**, 184 P 0.991**, 191 P 1.000**, 193 C 0.998**, 202 C 0.997**, 662 S 0.999**, 667 A 0.981*, 668 F 0.991**, 669 L 0.992**, 671 P 0.987*, 681 I 0.976*, 683 T 0.999**, 686 I 1.000**, 689 I 1.000****
Hspa12a	0.05343	8 S 0.919
Hspa12b1	**0.0000**	**627 K 0.999**, 628 A 1.000**, 629 V 0.999**, 630 A 0.954*, 631 S 0.957***, 632 L 0.943, **635 G 0.999****, 636 P 0.926, **645 A 0.999**, 647 G 0.982*, 648 V 0.979*, 650 L 0.954***, 657 E 0.942, **660 L 0.999**, 662 Q 0.999**, 664 F 0.982*, 665 G 1.000**, 666 E 0.999****, 668 F 0.931, **669 I 0.999****, 670 Q 0.941, **671 S 1.000**, 673 K 0.979***, 675 K 0.914, **679 A 0.965*, 685 L 0.975*,**

		689 F 0.911, **691 A 0.999****, 693 K 0.919, **694 R 1.000****, 695 T 0.934, 697 A 0.922, **698 P 1.000****, **699 G 1.000****, **700 R 1.000****, **701 A 0.971***, 703 A 0.906, **707 S 1.000****, **708 L 0.984***, **709 P 1.000****, **711 S 0.971***, **712 F 0.999****, **715 Y 0.999****, **716 Y 0.987***, **717 K 1.000****, **718 R 1.000****, **720 R 0.984***, **723 S 0.988***, **724 V 0.999****, **725 E 1.000****, **727 A 0.998****, **729 R 1.000****, **730 R 0.996****, 732 K 0.922
Hspa12b2	**0.0000**	**32 P 0.958***, 36 T 0.918, 39 V 0.935, 41 L 0.907, 43 G 0.923, 46 P 0.936, 49 R 0.906, 112 C 0.903, **321 D 0.995****, **323 T 1.000****, 327 I 0.907, **339 K 0.999****, **340 A 0.998****, 341 S 0.929, **343 E 0.999****, **344 L 0.963***, **346 A 1.000****, **347 K 1.000****, **351 R 0.998****, **353 V 0.999****, **355 F 0.997****, **366 P 0.999****, **367 M 0.997****, **368 L 0.998****, **370 K 0.999****, **371 A 0.998****, **372 V 0.963***, **374 K 1.000****, **375 A 0.999****, **377 G 0.999****, **379 T 1.000****, **384 I 0.963***, **408 S 1.000****, **409 Q 1.000****, **411 H 0.997****, **418 L 1.000****, **419 F 0.866**, **420 D 0.997****
Hspa13	**0.0039**	/
Hspa14	0.4162	143 K 0.959*
Hsc70	**0.0000**	**431 C 0.964***, 432 R 0.926, 433 T 0.935, **434 H 0.999****, 1040 F 0.929, **1042 G 1.000****, 1118 V 0.910, **1120 P 1.000****, **1126 S 0.999****, **1130 T 1.000****, **1134 S 1.000****, **1136 N 0.996****, **1137 K 0.998****, 1140 T 0.937, **1193 Q 0.987***, 1195 D 0.937, **1197 G 0.997****, **1202 L 1.000****, **1204 C 1.000****
Hyou1	0.2306	/
Hsp90aa1.1	1.0000	/
Hsp90aa1.2	**0.0000**	16 G 0.908, 22 D 0.916, **431 M 0.978***, **432 H 0.996****, **488 D 0.974***, **489 M 0.982***, **491 F 1.000****, **492 V 0.974***
Hsp90ab1	**0.0000**	**444 R 1.000****, 445 L 0.904, **446 T 0.998****, **447 K 1.000****, **459 K 1.000****, **460 L 0.999****, 500 K 0.942, **501 W 0.998****, **502 I 0.998****, **503 R 0.993****, **504 R 0.994****
Hsp90b1	**0.0000**	**723 S 0.983***, **791 D 0.998****, **965 H 0.981***, 1099 E 0.940
Trap1	0.0703	**564 H 0.970***
Dnajb4	0.661 3	239 N 0.861
Dnajb5_10016268	1.000 0	289 Y 0.507
Dnajb11	1.000 0	/
Dnajb12	1.000 0	/
Dnajc5ga	1.000 0	/
Dnajc9_10000949	**0.007 9**	77 R 0.825,**95 K 0.961***,**96 E 0.990***,97 A 0.522,**109 V 0.978***,**130 V 0.980***,141 K 0.930,204 V 0.921,205 Q 0.896,206 H 0.944,207 Q 0.791,211 D 0.921,216 S 0.596,**220 C 0.970***,244 F 0.710,269 A 0.614,276 M 0.650,283 D 0.911,286 V 0.972*,318 S 0.661,**320 D 0.976***,**338 N 0.994****,350 E 0.700
Dnajc9_10009927	1.000 0	/
Dnajc22	1.000 0	/
