# Supplementary figures and images for: Genomic and Transcriptomic Landscape and Evolutionary Dynamics of Heat Shock Proteins in Spotted Sea Bass (Lateolabrax maculatus) under Salinity Change and Alkalinity Stress
Source: Biology (Basel). 2022 Feb 23;11(3):353. doi: 10.3390/biology11030353 (PMC8945262; doi:10.3390/biology11030353)

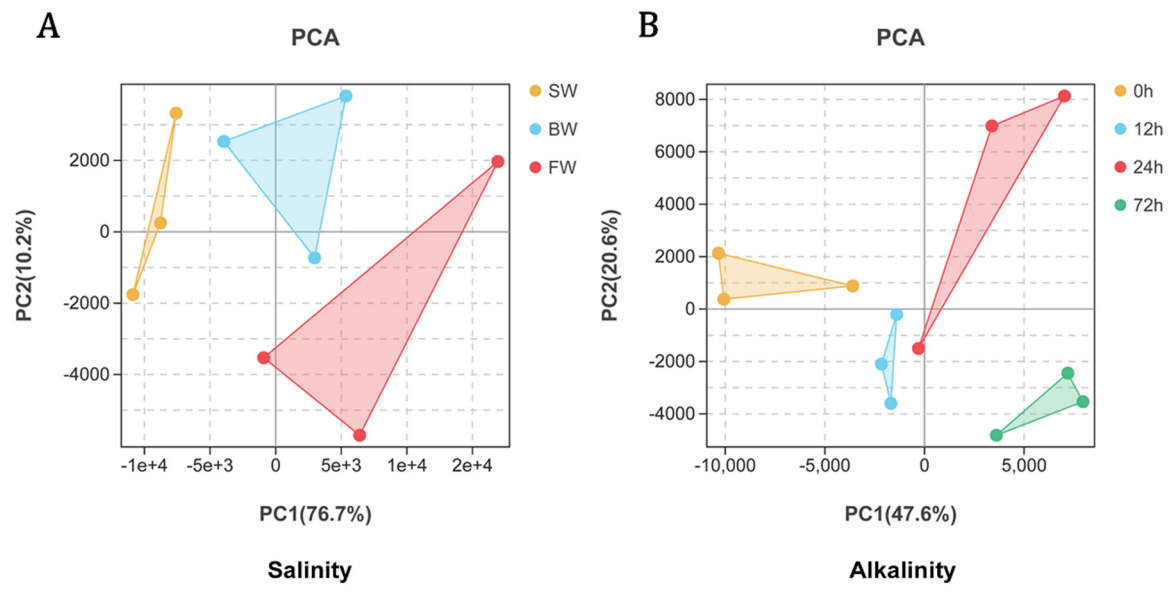

Supplement: Supplementary file 1 [file biology-11-00353-s001.zip › Figure S1. PCA for Salinity and Alkalinity.pdf]

**A**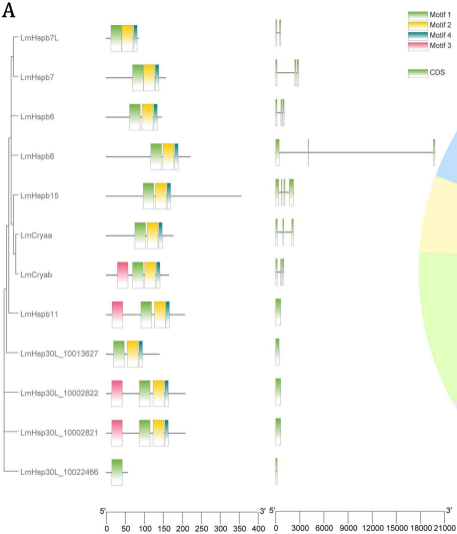

**B**

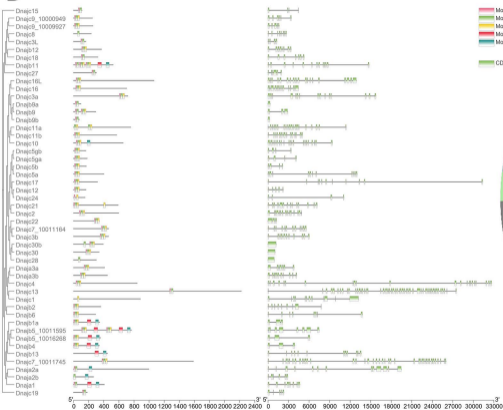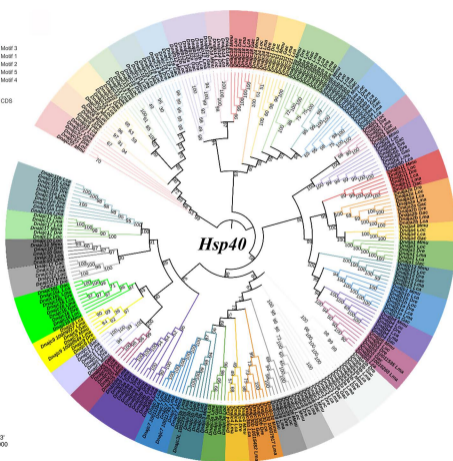

**C**

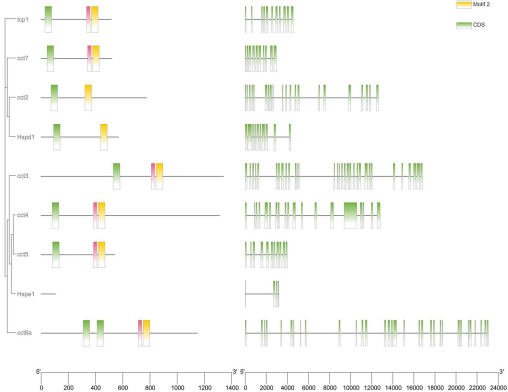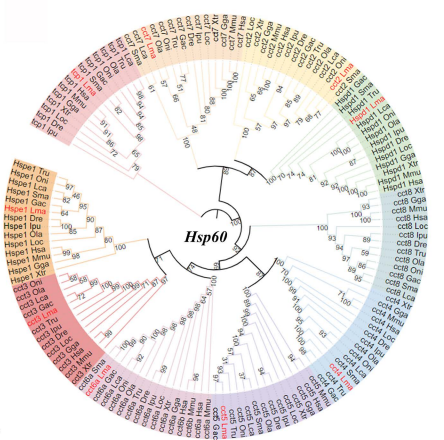

D

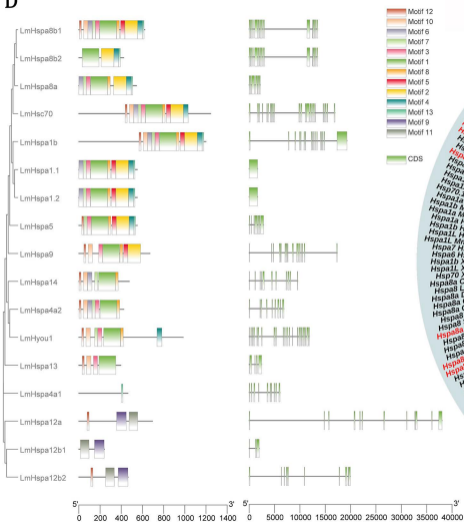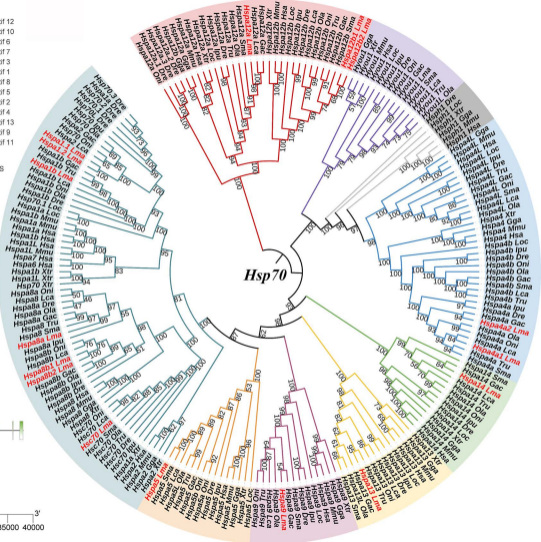

Supplement: Supplementary file 1 [file biology-11-00353-s001.zip › Figure S2. Phylogeny of LmHsps.pdf]

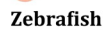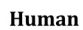

Supplement: Supplementary file 1 [file biology-11-00353-s001.zip › Figure S3. PPI for Zebrafish and Human.pdf]
